# Strategies for the Prevention of Invasive Fungal Infections after Lung Transplant

**DOI:** 10.3390/jof7020122

**Published:** 2021-02-07

**Authors:** Roni Bitterman, Tina Marinelli, Shahid Husain

**Affiliations:** Transplant Infectious Diseases, Ajmera Transplant Center, University Health Network, University of Toronto, Toronto, ON M5G 2C4, Canada; roni.bitterman@uhn.ca (R.B.); tina.marinelli2@uhn.ca (T.M.)

**Keywords:** lung transplantation, invasive fungal disease, invasive aspergillosis, prophylaxis, preemptive treatment

## Abstract

Long-term survival after lung transplantation is lower than that associated with other transplanted organs. Infectious complications, most importantly invasive fungal infections, have detrimental effects and are a major cause of morbidity and mortality in this population. *Candida* infections predominate in the early post-transplant period, whereas invasive mold infections, usually those related to *Aspergillus*, are most common later on. This review summarizes the epidemiology and risk factors for invasive fungal diseases in lung transplant recipients, as well as the current evidence on preventive measures. These measures include universal prophylaxis, targeted prophylaxis, and preemptive treatment. Although there is consensus that a preventive strategy should be implemented, current data show no superiority of one preventive measure over another. Data are also lacking regarding the optimal antifungal regimen and the duration of treatment. As all current recommendations are based on observational, single-center, single-arm studies, it is necessary that this longstanding debate is settled with a multicenter randomized controlled trial.

## 1. Epidemiology

Despite advances in donor selection, surgical techniques, and immunosuppressive regimens, long-term survival after lung transplantation has only slightly improved throughout the last two decades, and the 5-year survival post-transplant is still around 50%–60% [1]. Invasive fungal diseases (IFDs) significantly contribute to the mortality seen among lung transplant recipients (LTRs) [2,3,4,5]. In a single-center study in the US, IFD was identified as the strongest predictor for mortality in these patients [6]. However, studies differ in terms of the time points reported for mortality and the definition of IFD used (some reporting on all IFDs, some on invasive mold diseases (IMDs), and some on invasive aspergillosis only). They all have a high mortality rate in common, ranging from 21.7% three months after the diagnosis of IFD to 58% two years after the diagnosis [2,4].

LTRs are especially prone to fungal infections due to the combination of intense immune suppression, continuous exposure of the transplanted organ to the environment, a depressed cough reflex, and airway anatomical abnormalities. The Transplant Infection Surveillance Network (TRANSNET) reported that among data collected from 11 US transplant centers between 2001 and 2006, 8.6% of LTRs in the first year post-transplant developed an IFD. Most of these infections were IMDs (5.5%), most commonly, invasive aspergillosis [4,7]. Other studies differ in the duration of follow-up and the fungal infection reported on; however, more extensive series reported an IMD rate of 5.4–9% [2,5,8,9]. In the immediate postoperative period (up to 30 days), *Candida* infections predominate, whereas later on, mold infections are more common [7,10]. Aside from *Aspergillus*, which is undoubtedly the most common mold isolated, other molds include zygomycetes, *Scedosporium*, and dematiaceous molds, and the incidence varies between studies [4,11].

Most studies focused on IFD in the first 6–12 months post-transplant, as this was thought to be the most vulnerable time window [7,9]. Recently, there have been studies emphasizing the importance of late-onset IFDs. A study conducted in Ontario, Canada, demonstrated that among 942 LTRs between 2002 and 2016, the median time to the first diagnosis of IFD was 1.5 years. In that study, there was a 7.4% incidence of IFD in the first year post-transplant and a 17.2% cumulative prevalence of IFD [3]. Another study from Toronto showed that among 350 LTRs that survived beyond the first year post-transplant, 9% developed invasive aspergillosis in the second to fourth-year post-transplant [5]. Non- *Aspergillus* molds, most notably *Scedosporium* spp. and zygomycetes, tend to appear later in the post-transplant course and are associated with a worse prognosis than invasive aspergillosis [12].

## 2. Risk Factors

Invasive mold infections are primarily acquired via the inhalation of fungal spores and conidia. On the one hand, the transplanted allograft has continuous contact with the environment and fungal elements of ubiquitous fungi, such as *Aspergillus*. On the other hand, LTRs have an impaired cough reflex and impaired mucociliary clearance due to the surgery. This combination puts LTRs at an exceptionally high risk for IMD. Numerous studies have evaluated risk factors for IFD and IMD among LTRs, with most of them being single-center studies. Amongst all risk factors, it seems that post-transplant fungal colonization is the most potent risk factor for IMD, as shown in several small single-center studies demonstrating an odds ratio (OR) of 6.69–22.4 for invasive aspergillosis [13,14,15]. A more significant multi-center cohort comprising 900 LTRs revealed a more modest association between 1-year post-transplant *Aspergillus* colonization and invasive disease, with an OR of 2.11 (95% CI 1.28–3.49) [16]. Pre-transplant *Aspergillus* colonization, a single-lung transplant, rejection and augmented immune suppression, age, ischemia time, early airway ischemia, daclizumab induction treatment, re-transplant, diabetes, renal replacement treatment, the performance status, CMV infection, and hypogammaglobulinemia have been inconsistently shown to be associated with an increased risk for IMD in single-center studies [2,8,17,18,19,20]. However, a large multicenter study found, using a multivariate analysis, that only a single-lung transplant (OR 1.84, 95% CI 1.09–3.1) and *Aspergillus* colonization less than one-year post-transplant (OR 2.11, 95% CI 1.28–3.49) were significantly associated with invasive aspergillosis [16]. Cystic fibrosis seems to have a strong association with IMD, mainly through pre- and post-transplant *Aspergillus* colonization. Several studies demonstrate high tracheobronchial aspergillosis rates and invasive aspergillosis in these patients [21,22,23].

## 3. Diagnosis

The ISHLT guidelines define fungal colonization as the presence of fungus in respiratory secretions identified by a culture, polymerase chain reaction (PCR), or biomarker in the absence of symptoms, and radiological and endobronchial changes [19]. Identifying fungal growth in a respiratory culture is still regarded as the gold standard for diagnosing fungal infection. It has the advantage of allowing the performance of susceptibility testing and identifying non-*Aspergillus* molds that would not be identified otherwise. On the other hand, the sensitivity is only approximately 50% [24,25], limiting its usefulness. Other diagnostic methods, mainly PCR and fungal biomarkers, offer an improved sensitivity when combined with a culture and address the limitations of conventional culture methods. The use of PCR is hampered by the lack of standardization between the different tests. However, a meta-analysis, mostly including patients with hematological malignancies, demonstrated a pooled sensitivity and specificity rate of 90.2% and 96.4%, respectively, for *Aspergillus* PCR in bronchoalveolar lavage (BAL) [26]. Of the available fungal biomarkers, galactomannan is the one most widely used. The Platelia galactomannan enzyme immunoassay is the only test used worldwide and is thus highly standardized. The test identifies carbohydrate residues in the *Aspergillus* cell wall. As galactomannan is also present in the cell wall of *Fusarium*, *Histoplasma*, *Blastomyces*, and *Penicillium*, there can be false positives [27]. Serum galactomannan should not be used in LTRs due to its poor performance. A meta-analysis revealed a pooled sensitivity rate of 22% for proven invasive aspergillosis and 41% for probable invasive aspergillosis in the solid organ transplant population [28]. BAL galactomannan testing has a far better diagnostic value, with sensitivity rates ranging from 60% to 82% in the solid organ transplant population [29,30]. BAL galactomannan can also be assessed using a lateral flow assay, shortening the turnaround time considerably. In a study including 38 solid organ transplant patients, most of whom were LTRs, the test’s sensitivity and specificity, using a 1 optical density index as the cutoff, were 100% and 42%, respectively. Increasing the cutoff to a 1.5 optical density index yielded a sensitivity and specificity of 78% and 67%, respectively, demonstrating results that are more comparable to the diagnostic values among patients with hematological malignancies [31]. (1-3)-β-d-glucan is a cell wall component in most fungi, except for zygomycetes, which do not produce it, and *Cryptococcus*, which only produces a small amount. It is thus very non-specific, as shown in two studies evaluating (1-3)-β-d-glucan in serum [32] and BAL [33] among LTRs for the diagnosis of invasive aspergillosis. The first study demonstrated a specificity of 9% for serum (1-3)-β-d-glucan when using a per-patient analysis and 59% in a per-test analysis [32]. The study evaluating BAL (1-3)-β-d-glucan also exhibited a low specificity ranging from 53 to 70%, depending on the cutoff used [33]. Due to this low to moderate specificity, (1-3)-β-d-glucan is not usually used for diagnosing fungal infections in LTRs.

## 4. Prevention Strategies

### 4.1. Definitions of Prophylaxis Strategies

**Figure 1:**
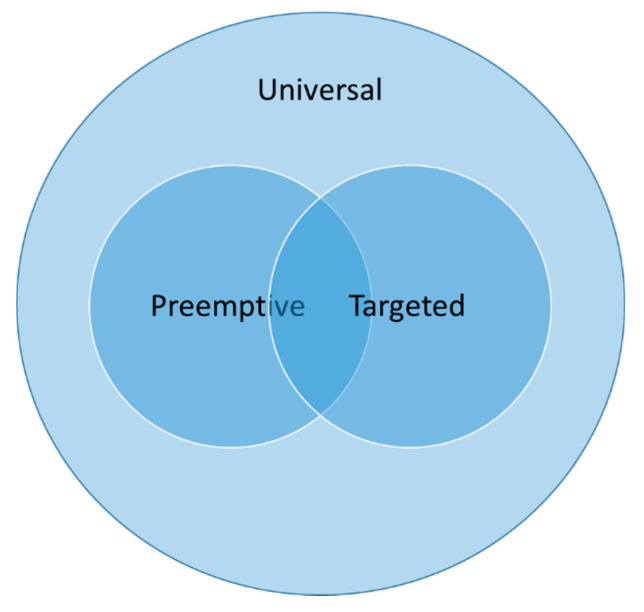
Invasive fungal disease prevention strategies employed in lung transplant recipients.

In their 2015 guidelines for fungal infections in cardiothoracic transplant recipients, The International Society for Heart and Lung Transplantation (ISHLT) defines the three strategies used to prevent fungal infections in this population [19]. Universal antifungal prophylaxis refers to administering antifungal treatment for all LTRs before the isolation of a fungal pathogen in the postoperative period. Targeted prophylaxis refers to the administration of antifungal treatment in the postoperative period before there is a fungal pathogen isolated in the postoperative period, but only to LTRs at high risk of fungal infection (e.g., pre-transplant fungal colonization, cystic fibrosis, etc.). Preemptive treatment refers to only administering antifungal treatment in the postoperative period to LTRs with fungal colonization and without evidence of IFD (Figure 1).

### 4.2. Current Practice and Recommendations

In recent years, it seems that more transplant centers have been using prophylaxis strategies over preemptive treatment. Pennington et al. conducted a retrospective study based on administrative claims data of commercial and Medicare Advantage enrollees in the US, and found that in the years 2005–2007, the ratio of LTRs receiving prophylaxis to those not receiving it was 0.99, and in the years 2015–2018, the ratio was 1.99 [34]. A multinational survey from 2009 included data from 58 centers worldwide and showed that 58.6% of centers implement a strategy of universal prophylaxis [35] compared to 78% in a survey of 27 US centers from 2013 [36]. It was also demonstrated that, whereas earlier surveys showed that the most common antifungals were nebulized amphotericin B deoxycholate (nAmBd) and itraconazole [37], more recent surveys demonstrate an increased use of nebulized liposomal preparations of amphotericin B and voriconazole [35,36]. In recent years, there have also been publications on the use of isavuconazole and posaconazole [38,39]. The duration of prophylaxis varied greatly between centers, ranging from less than three months to more than one year (and probably lifelong) [36]; however, it seems that most centers use prophylaxis for up to six months [35].

The Infectious Diseases Society of America (IDSA), the Europeans Society of Clinical Microbiology and Infectious Diseases (ESCMID), the American Society of Transplantation (AST), and the ISHLT have all recently issued guidelines addressing the prevention of fungal infections in LTRs. The AST [40] and ISHLT [19] guidelines, published in 2019 and 2016, respectively, both provide the option of using either universal prophylaxis or preemptive treatment, acknowledging that the quality of evidence is low. They recommend using either nAmB or a mold-active azole for universal prophylaxis, but only a mold-active azole for preemptive treatment. Suggested treatment durations are 4–6 months for prophylaxis and 3–4 months for preemptive treatment. The IDSA guidelines [27] published in 2016 and the ESCMID guidelines [41] published in 2018 both recommend universal prophylaxis as the preferred method for preventing IFD. IDSA guidelines recommend prophylaxis with either nAmB or a mold-active azole for 3–4 months post-transplant. They also recommend reinitiating prophylaxis during periods of augmented immune suppression. For patients with either pre- or post-transplant mold colonization, molds in the explanted lung, or a single-lung transplant, it is recommended that only mold-active azoles are applied [27].

### 4.3. Current Data on Prevention Strategies

We performed a comprehensive search of the literature using Medline database from inception to December 2020 and identified 28 studies evaluating different strategies for preventing fungal disease in LTRs (Table 1). Fifteen studies were single-arm studies assessing universal prophylaxis (10 studies) [42,43,44,45,46,47,48,49,50,51], universal and targeted prophylaxis (2 studies) [8,52], preemptive treatment and targeted prophylaxis (2 studies) [53,54], and no preventive strategy (1 study) [55]. Thirteen studies were comparative, comparing universal prophylaxis with no preventive strategy (5 studies) [34,56,57,58,59], universal prophylaxis with preemptive treatment (2 studies) [13,16], universal prophylaxis with targeted prophylaxis (2 studies) [10,60], and different drugs for universal prophylaxis (4 studies) [38,61,62,63]. Only one study was a randomized controlled trial [61], whereas all the others were observational trials. The studies identified included data from the years 1990 to 2017. The duration of antifungal prophylaxis and/or treatment ranged from only during the transplant-associated hospitalization to lifelong antifungal prophylaxis. The duration of follow-up varied from two months to 60 months. All studies reported on either IFD or invasive aspergillosis; however, only 13 studies reported fungal colonization and mortality.

**Figure 2:**
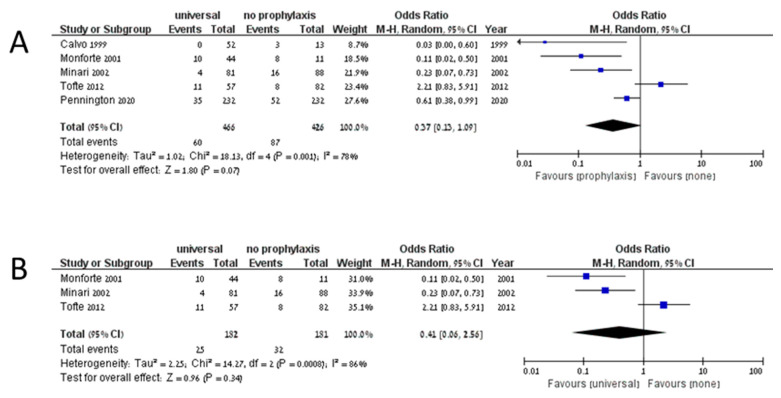
Invasive fungal disease (**A**) and invasive aspergillosis (**B**) in studies comparing universal prophylaxis and no prophylaxis.

**Figure 3:**
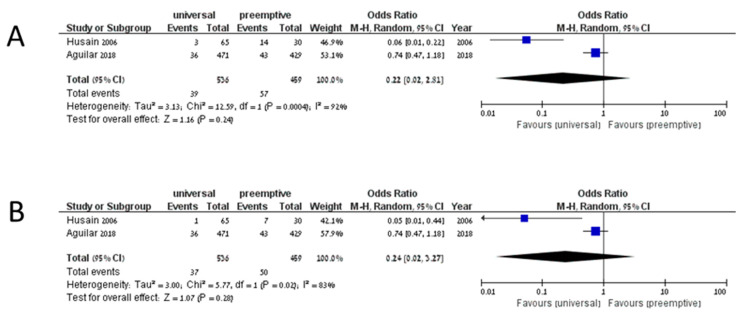
Invasive fungal disease (**A**) and invasive aspergillosis (**B**) in studies comparing universal prophylaxis and preemptive treatment.

The cumulative incidence of IFD among 3561 patients on universal prophylaxis was 0.099 (95% CI 0.074–0.132, I^2^ 83.9%), whereas the cumulative incidence of invasive aspergillosis among 3329 patients was 0.063 (95% CI 0.047–0.084, I^2^ 68%). In studies assessing preemptive treatment, the cumulative incidence of IFD was 0.121 (95% CI 0.035–0.345, I^2^ 90.5%), and the cumulative incidence of invasive aspergillosis was 0.097 (95% CI 0.044–0.199, I^2^ 67.8%). No comparative analysis yielded significant results in favor of any preventive strategy. Five studies compared universal prophylaxis with no prophylaxis [34,56,57,58,59]. Of 466 patients on universal prophylaxis, 60 developed IFD compared to 87 of 426 patients that developed IFD without prophylaxis (OR 0.37, 95% CI 0.13–1.09, I^2^ 78%, Figure 2A). The odds ratio for invasive aspergillosis in patients on universal prophylaxis compared to no prophylaxis was 0.41 (95% CI 0.06–2.56, I^2^ 86%, Figure 2B). Only two studies compared universal prophylaxis with preemptive treatment [13,16]. Among patients on universal prophylaxis, 39/536 developed IFD compared to 57/459 on preemptive treatment (OR 0.22, 95% CI 0.02–2.81, I^2^ 92%, Figure 3A), and 37/536 compared to 50/459 developed invasive aspergillosis (OR 0.24, 95% CI 0.02–3.27, I^2^ 83%, Figure 3B). When comparing universal prophylaxis to either targeted prophylaxis or preemptive treatment, we identified four studies [10,13,16,60] and found no significant difference between the strategies (OR for IFD 0.66, 95% CI 0.14–3.06, I^2^ 90%). Since only 13 studies included data on mortality and studies differed in the time point on which mortality was reported, we could not compile the data.

As shown in the analyses above, no prevention strategy is superior to another. Most available data are derived from small single-center, single-arm studies, explaining the very high heterogeneity observed. Only the analysis comparing universal prophylaxis to no prophylaxis almost reached statistical significance (OR 0.37, 95% CI 0.13–1.09). This observation is in agreement with common practice in which a preventive strategy is implemented.

Three systematic reviews and meta-analyses have been published to date on this subject [64,65,66]. Two of the systematic reviews reached a similar conclusion. However, one systematic review concluded that universal prophylaxis is superior [66]. This systematic review included fewer studies. The studies were not as recent as the studies included here (considering that two of the largest series have only recently been published [8,16]). In this systematic review, universal prophylaxis was compared to all other strategies combined (targeted prophylaxis, preemptive treatment, and no prophylaxis). Nevertheless, we believe that no prophylaxis (which is not routinely practiced) cannot be grouped with other preventive strategies. Therefore, universal prophylaxis’s superiority should not be inferred from the systematic review published by Pilarczyk et al. [66].

### 4.4. Choice of Drug

Both mold-active azoles and nAmB preparations have been used for the prevention of IFD in LTRs. However, studies directly comparing different drugs and using the same prevention strategy are lacking. Two studies, of which one was a randomized controlled trial, compared universal prophylaxis with nAmBd to nebulized lipid formulations of AmB [61,63]. Combined results demonstrated no difference in risk for IFD (OR 1.43 95% CI 0.52–3.93, I^2^ 0%). A small study compared universal prophylaxis with itraconazole to voriconazole and demonstrated a nonsignificant trend towards a better efficacy with voriconazole [62]. This could possibly be secondary to the inferior pharmacokinetic-pharmacodynamic profile of itraconazole. With the advent of newer mold-active azoles, itraconazole has fallen out of favor. A recently published trial on 300 LTRs demonstrated a similar efficacy of voriconazole and isavuconazole in terms of preventing IFD [38]. In summary, it seems that both nAmB and mold-active azoles are adequate agents for universal prophylaxis. As nAmB has not been evaluated for targeted prophylaxis or preemptive treatment, it should not be used for these indications.

### 4.5. Duration

The duration of antifungal treatment in studies evaluating universal prophylaxis ranged from only during the initial hospitalization [10,44] to lifelong prophylaxis [50,63]. Since most studies gave prophylactic antifungals for up to six months, coinciding with the highest risk for IFD [4,16], it seems reasonable to limit universal prophylaxis to that period. Studies assessing preemptive treatment administered antifungals for 3–6 months [13,53,54]. Taking this into account, together with the recommended treatment duration for invasive aspergillosis [27], a treatment duration of approximately three months is suggested for preemptive treatment.

### 4.6. Toxicity

The use of nAmB preparations is associated with respiratory side effects, such as a cough, shortness of breath, and bronchospasm, and gastrointestinal side effects, mainly nausea and vomiting. One randomized trial that compared nAmBd to a nebulized amphotericin B lipid complex (nABLC) showed the significantly better tolerability of nABLC [61]. Nonetheless, the discontinuation of prophylaxis due to side effects did not differ between the two groups, although the study was underpowered for that. When combining the results with a second study that compared prophylaxis with nAmBd to nebulized liposomal amphotericin B (nLAmB) [63], there was no difference in the rate of discontinuation due to side effects (OR 1.88 95% CI 0.61–5.84).

Mold-active azoles, mainly itraconazole and voriconazole, are associated with numerous side effects and drug–drug interactions [27]. Azoles can cause gastrointestinal intolerance, hepatotoxicity, neurotoxicity, and prolongation of the QT interval, although the probability varies which each drug. Itraconazole exhibits significant variation in absorption, and its most problematic side effect is gastrointestinal intolerance. The main side effects of voriconazole and limiting its use are significant hepatotoxicity, visual disturbances, and numerous drug–drug interactions. Long-term treatment is also associated with an increased risk of skin cancer [27]. A retrospective study on 193 LTRs from Mayo Clinic, Rochester, found that 68.8% of patients on voriconazole prophylaxis discontinued treatment. In over half of the cases, it was due to side effects or intolerance. Additionally, 61.8% of patients on itraconazole prophylaxis discontinued treatment, most commonly due to malabsorption (15.7%) and suspected breakthrough infection (10.2%). Contrarily, only 18.3% of patients on posaconazole prophylaxis had to discontinue treatment [39]. Another recently published study found voriconazole and isavuconazole prophylaxis to be equally effective, although the discontinuation rate was significantly higher for voriconazole (36% vs. 11%), mainly due to hepatotoxicity [38]. The drug toxicities of commonly used antifungals are summarized in Table 2.

## 5. Conclusions

IFD, most commonly invasive aspergillosis, remains a significant cause of morbidity and mortality among LTRs. Although it seems that a preventive measure is beneficial, current evidence does not support any specific preventive strategy. The strengths and weaknesses of the different strategies are summarized in Table 3. There is also insufficient evidence to endorse the use of one drug over another. After more than two decades of numerous observational studies, it is time we obtain a definitive answer. A multi-center randomized controlled trial is thus urgently needed.

## Figures and Tables

**Table 1:** Studies evaluating preventive strategies for fungal disease in lung transplant recipients.

Study	Years	N	Strategy	Antifungal	Duration of Prophylaxis, m	Duration of Follow-up, m	IFD	IA	Colonization (*Aspergillus*/mold)	Mortality
Non-comparative studies										
Hamacher 1999	1993–1997	31	Preemptive + targeted ^^^	Itraconazole	4.2, mean	19.4, mean	3 (9.6%)	2 (6.4%)		8 (25.8%)
Palmer 2001	1997–1998	51	Universal	nABLC	2	12 ^~^	6	0		
Shitrit 2005	1994–2004	40	Universal	Itraconazole	6	12	2 (5%)	2 (5%)	11 (27.5%)	
Lowry 2007	2002–2004	38	Universal	nAmBd/nLAmB	0.25, median	NS	1 (2.6%)	1 (2.6%)	0	
Borro 2008	2005–2007	60	Universal	nABLC + fluconazole	nABLC-3m fluconazole-3w	6	0	0	1 (0.15%)	
Eriksson 2010	2002–2010	76	Universal + targeted ^#^	Universal-nAmBd/nABLB Targeted-caspofungin	Universal-till anastomosis heals Targeted-not specified	31.2, median	3 (3.9%)	3 (3.9%)	12 (15.7%)	11 (14.5%)
Hayes 2011	2001–2005	41	Universal	Itraconazole	12, median	Varied, at least 12	8 (19.5%)	6 (14.6%)		32% ^@^ 3y
Pinney 2011	1994–2006	242	None			34, median	22 (9%)	11 (4.5%)		44% ^@^ 3y
Mitsani 2012	2009	93	Universal	Voriconazole	At least 3	NS	10 (10.7%)	1 (1%)	6 (6.4%)	
Neoh 2013	NS	62	Preemptive	Voriconazole	3, median	12	1 (1.6%)	1 (1.6%)		16 (25.8%)
Kato 2014	2008–2012	30	Universal	Itraconazole ^~^	Variable, at least 12	60 ^~^	5 (16.6%)	5 (16.6%)		
Chong 2015	2002–2011	91	Universal	Voriconazole/itraconazole ^@^	At least 12 (most lifelong)	Variable, at least 12	15 (16.5%)	10 (10.9%)	27 (29.6%)	15.3% @ 1y 49.4% ^@^ 3y
Peghin 2016	2003–2013	412	Universal	nLAmB	Lifelong	30.7, mean	22 (5.3%)	22 (5.3%)	61 (14.8%)	
Stelzer 2018	2014–2016	9	Universal	Posaconazole	At least 6	15, median	0	0	2 (22.2%)	
Baker 2020	2007–2014	815	Targeted *	Universal-nABLC Targeted-mold-active azole/micafungin/nABLC	Universal-till discharge Targeted-variable	3	156 (19.1%)	42 (5.1%)		
Comparative studies										
Calvo 1999	1990–1997	52	Universal	nAmBd + fluconazole	Till discharge 1.4, mean	During hospitalization	0	0		
		13	None				3 (23%)	NS		
Monforte 2001	1990–1997	44	Universal	nAmB	Lifelong	14, mean	10 (22.7%)	10 (22.7%)	12 (21.8%, combined)	23 (41.8%, combined)
		11	None				8 (72.7%)	8 (72.7%)		
Minari 2002	1990–1999	81	Universal	nAmBd + itraconazole	nAmBd-post-transplant itraconazole- lifelong?	variable	4 (4.9%)	4		
		88	None				16 (18.1%)	16		
Drew 2004 RCT	1999–2002	49	Universal	nAmBd	7w	2	7 (14.2%)	1 (2%)		
		51	Universal	nABLC	7w		6 (11.7)	1 (2%)		
Matter 2005	2002–2003	18	Targeted ^$^	Voriconazole	6w	During hospitalization	1 (5.6%)	1		
		101	Universal	Itraconazole	NS		6 (5.9%)	6		
Husain 2006	2001–2004	65	Universal	Voriconazole	≥4	12	3 (4.6%)	1 (1.5%)	16 (24%)	2 (3%)
		30	Universal + preemptive ^&^	Fluconazole-universal Itraconazole ± nAmBd- preemptive	3–6		14 (46.6%)	7 (23.3%)	12 (40%)	5 (16%)
Cadena 2009	2003–2006	32	Universal	Itraconazole	≥3	12	4 (12.5%)	4	11(34.3%)	4 (12.5%)
		35	Universal	nAmBd + voriconazole	≥3 (nAmBd-2w)		1 (2.8%)	0	9 (25.7%)	7 (20%)
Monforte 2010	2000–2001	49	Universal	nAmBd	Lifelong	12	2 (4.1%)	2 (4.1%)	1 (2%)	
	2003–2005	104	Universal	nLAmB	Lifelong		2 (1.9%)	2 (1.9%)	5 (4.8%)	
Koo 2012	2003–2010	82	Universal	nAmBd/nLAmB	During hospitalization	12	29 (35.3%)	8		13 (16%)
		83	Universal + targeted ^%^	nAmb + micafungin ± tailored antifungal	nAmB-during hospitalization micafungin- 10d tailored antifungal- 3–6		10 (12%)	2		9 (11%)
Tofte 2012	2002–2006	57	Universal	Voriconazole	3	Up to 60	11 (19.2%)	11 (19.2%)	12 (21%)	7% @ 1y 21% @ 3y
		82	None				8 (10%)	8 (10%)	23 (28%)	29% @ 1y 43% @ 3y
Aguilar 2018	2005–2008	471	universal	Mostly nAmB or itraconazole	Variable	48, median	36 (7.6%)	36 (7.6%)		
		429	Targeted/preemptive	Variable	Variable		43 (10%)	43 (10%)		
Samanta 2020	2013–2015	144	Universal	Isavuconazole (+nAmB 100%)	3.4, median	At least 12	10 (6.9%)	3 (2%)	19 (6%)	14 (10%)
		156	Universal	Voriconazole (+nAmB 41%)	3.1, median		13 (8.3%)	7 (4.4%)	5 (3%)	18 (12%)
Pennington 2020	2002–2017	232	Universal + targeted	Variable	Variable	12	14.94% (adjusted)			8.36% (adjusted)
		232	none				22.37% (adjusted)			19.49% (adjusted)

Abbreviations: N, number; m, months; w, weeks; y, years; d, days; IFD, invasive fungal disease; IA, invasive aspergillosis; nAmB, nebulized amphotericin B; nAmBd, nebulized amphotericin B deoxycholate; nABLC, nebulized amphotericin B lipid complex; nLAmB, nebulized liposomal amphotericin B; NS, not specified. ^^^ Positive pre- and post-transplant cultures. ^#^ CF, elderly + single lung transplant, pre-transplant colonization, explant mycetoma, necrotizing tracheobronchial aspergillosis, and suspected mediastinal contamination. ^~^ Micafungin till itraconazole levels achieved. ^@^ Capofungin/fluconazole till oral intake tolerated. * Pre-transplant mold colonization/alemtuzumab, mold-active azole; delayed chest closure/ECMO, mica; acute cellular rejection, nABLC; post-transplant colonization, variable. ^$^ Pre-transplant *Aspergillus* colonization or unexplained perioperative fever. ^&^ Pre- or post-transplant *Aspergillus* colonization (except *A. niger*). ^%^ Positive perioperative culture (mold or yeast).

**Table 2:** Toxicities of antifungals used for prophylaxis and preemptive treatment.

Drug	Short-Term Toxicity	Long-Term Toxicity
Nebulized Amphotericin B	Respiratory-cough, shortness of breath, bronchospasm	Damage to surfactant causing deterioration in pulmonary function (suspected)
	GI-nausea, vomiting	
Azoles Itraconazole	AKI secondary to cyclodextrin in IV formulation (suspected)	Neurologic-peripheral neuropathy
	DDI-CYP3A4 (inhibitor + substrate), Pgp (inhibitor)	
	GI-nausea, vomiting (most)	
	Hepatotoxicity	
	Prolongation of QT interval	
Voriconazole	AKI secondary to cyclodextrin in IV formulation (suspected)	Periostitis
	DDI-CYP2C19 (inhibitor + substrate), CYP3A4 (inhibitor + substrate)	Peripheral neuropathy
	Neurologic-visual disturbances, hallucinations	Alopecia
	GI-nausea, vomiting, diarrhea	Squamous cell carcinoma of the skin
	Hepatotoxicity (most)	
	Skin-rash, photosensitivity, perioral excoriations	
	Prolongation of QT interval	
Posaconazole	AKI secondary to cyclodextrin in IV formulation (suspected)	Not reported
	DDI-CYP3A4 (inhibitor)	
	GI-nausea, vomiting	
	Hepatotoxicity	
	Prolongation of QT interval	
Isavuconazole	DDI-CYP3A4 (inhibitor + substrate)	Not reported
	Hepatotoxicity	
	Shortening of QT interval	

Abbreviations: GI, gastrointestinal; AKI, acute kidney injury; DDI, drug–drug interactions.

**Table 3:** Pros and cons of the different prevention strategies.

	Pros	Cons
Universal prophylaxis	Easy to implement	Increased antifungal consumption Drives resistance Increased toxicity Increased drug–drug interactions
Preemptive treatment	Lower antifungal drug consumption	Requires resources (surveillance bronchoscopies, short turnaround time for galactomannan results)

## Data Availability

Not applicable.
